# 

**Published:** 1870-01

**Authors:** 


### The Dental Register

### OF THE WEST.

*Issued Monthly, at $3 a Year in Advance.*

DEVOTED TO THE INTERESTS OF THE DENTAL PROFESSION.

EDITED BY J. TAFT AND G. WATT.

The volume commences in January and closes in December.

The Register being issued monthly is always fresh, therefore indispensable to the practitioner who desires to keep posted up with the new inventions and improvements which are daily developed. Neither expense nor effort will be spared to give value and variety to its contents. Articles will be illustrated with well executed engravings whenever they will add to the interest or assist in explanation.

Its extensive circulation and frequency of issue makes it one of the BEST DENTAL ADVERTISING MEDIUMS in the United States.

RATES OF ADVERTISING.

One page, 1 year, $50. Half page, 1 year, $30. Quarter page, or card, 1 year $20.

All advertising bills payable in advance, or at furthest after the issue of the first number containing the advertisement. All transient advertisements to be paid for in advance

☞ CONTRIBUTIONS SOLICITED.

Address, J. TAFT, or "DENTAL REGISTER," Cincinnati Ohio.

Business Communications to

J. TAFT, Publisher,

No. 117 West Fourth St.

AMERICAN DENTAL ASSOCIATION—Meets on the first Tuesday in August next year at Nashville, Tenn. *Pres.* H. JUDD, St. Louis. *Rec. Sec.* M. S. DEAN, Chicago.

*List of Societies represented in the American Dental Association.*

AMERICAN DENTAL CONVENTION—Meets annually. *Pres.* J. M. CROWELL of New York, *Rec.* and *Cor Sec* J. S. LATIMER, of New York.

AMERICAN ACADEMY OF DENTAL SCIENCE—Meets at Boston, Mass. *Pres.* DANIEL HARWOOD, M. D., of Boston. *Sec.* E. N HARRIS, D. D. S., of Boston.

BROOKLYN DENTAL SOCIETY—Meets semi-monthly. *Pres.* C. D. Cook. *Rec. Sec.* E L. CHILDS. *Cor. Sec.* WM. JARVIE Jr.

BUCKS COUNTY DENTAL ASSOCIATION, (PA.) Meets semi-annually on the first Monday in May and November. Next meeting at Newton, in November. *Pres.* H. P. Yerkes, *Sec.* G. W. Adams.

BUFFALO DENTAL SOCIETY—Meets monthly at Buffalo. *Pres.* B. F. Whitney, *Rec. Sec.* S. A. FREEMAN.

BOSTON DENTAL INSTITUTE. Meets first Tuesday of each month. *Pres.* D. G. Williams. *Cor. Sec.* D S. Dickerman.

CUMBERLAND VALLEY DENTAL SOCIETY—Meets semi-annually.* *Pres.* J. L. SUESSEROTT, of Chambersburg. *Sec.* GEO. W NEIDICH, of Carlisle, Pa.

CINCINNATI DENTAL SOCIETY—Meets semi-monthly at Cincinnati. *Pres.* Geo. WATT, *Rec. Sec.* Wm. TAFT.

CENTRAL OHIO DENTAL ASSOCIATION—Meets Annually on the second Tuesday of July. *Pres.* S. P. HARRINGTON, Newark. *Sec.* G. W. DECAMP Mansfield, O.

CHICAGO DENTAL SOCIETY—Meets monthly at Chicago. *Pres.* J. H. YOUNG. *Rec. and Cor. Sec.* C. R E. KOCH.

CONNECTICUT VALLEY DENTAL ASSOCIATION—Meets semi-annually, second Tuesday of May and October, *Pres.* A. A. HOWLAND, Barre, Vt. *Rec. Sec* W. H. JONES, of Boston, Mass.

CONNECTICUT STATE DENTAL ASSOCIATION—*Pres.* SAML. MALLETT, New Haven. *Cor. Sec.* JOHN CODY, Hartford. *Rec. Sec.* Dr. POWERS, Hartford.

CHARLESTON DENTAL ASSOCIATION.*—*Prest.* I. B. PATRICK. *Sec. and Treas.* T. F. CHUPEIN.

DELAWARE DENTAL ASSOCIATION—Meets semi-annually. *Pres.* E, W. HAINES, Newark. *Sec.* C. R. JEFFRIES, Wilmington.

EAST TENNESSEE DENTAL ASSOCIATION—Meets annually at Knoxville, Tenn. *Pres* J. A. CRAZIER, of Knoxville *Rec. Sec.* W. F. FOWLER, Tenn.

FOREST CITY SOCIETY OF DENTAL SURGEONS *Pres.* B. Strickland, Cleveland. *Rec. Sec.*, H. L Ambler, Cleveland. *Cor. Sec.*, Chas. Buffett, Cleveland.

HARRIS DENTAL ASSOCIATION OF LANCASTER, Pa —Next meeting at Columbia, on Thursday the [illegible] th of Nov. next. *President*, SAM'L. WELCHENS. *Sec.* WM. NICHOLS AMER

HUDSON RIVER ASSOCIATION OF DENTAL SURGEONS—Meets on the second Wednesdays of May, September and January. *Pres.* L. S. STRAW, Newburg N.Y. *Rec. Sec.* T. W. DuBois, Pougkeepsie, N. Y.

HUDSON VALLEY DENTAL ASSOCIATION—meets monthly at Troy, N. Y. *Pres.* C. H. JENKINS, *Rec. Sec.* E. J. YOUNG, *Cor. Sec.* S. D. FRENCH.

ILLINOIS STATE DENTAL SOCIETY—Meets annually. *Pres.* M S. Dean, of Chicago. *Sec* C. S. SMITH, of Springfield.

INDIANA STATE DENTAL SOCIETY—Meets annually at Indianapolis on the last Tuesday of June. *Pres.* J. F. Johnston, of Indianapolis. *Rec. and Cor. Sec* S. B. Brown.

IOWA STATE DENTAL SOCIETY—Meets semi-annually Jan. and July. *Pres.* L. C. INGERSOLL of Keokuk. *Rec. Sec.* Wm. O. KULP, of Muscatine. *Cor. Sec.* N. H TULLOSS, of Iowa City.

LAKE ERIE DENTAL ASSOCIATION.—Meets Annually.* *Pres.* B.T. WHITNEY, Buffalo N. Y., *Sec.* W. E. MAGILL, Erie, Pa.

LEBANON VALLEY DENTAL ASSOCIATION—*Prest.* W. K. BRENIZER, *Sec.* S. H. GUILFORD.

MAD RIVER VALLEY DENTAL SOCIETY—Meets Semi annually on the first Tuesday of April and Oct. Next meeting at Springfield, O. *Pres.* J. H. PAINE, Middletown, O. *Sec.*, S. B. TIZZARD, Troy, O.

MARYLAND ASSOCIATION OF DENTISTS—Meets the last Thursday of every month. *Pres.*, J. B. BEAN, *Sec.*, S M. FIELD.

MASSACHUSETTS DENTAL SOCIETY—Meets monthly. *Pres.*, T. H. Chandler, *Rec. Sec* A. Brown, *Cor. See.* E. Blake.

MICHIGAN DENTAL SOCIETY—Meets annually second Tuesday of Oct. at Detroit. *Pres.* George Leary, of Adrian, *Rec. and Cor. Sec.* W. H. Jackson, of Ann Arbor.

MUSKINGUM VALLEY DENTAL ASSOCIATION—Meets on first Monday of each month at Zanesville, O. *Pres.* W. M. Herriot, of Zanesville, O., *Sec.* W. W. Chapelear, Zanesville, O.

MISSISSIPPI VALLEY DENTAL ASSOCIATION—Meets annually at Cincinnati, on the first Wednesday in March. *Pres.* A. M. Moore, Lafayette, Ind. *Rec. Sec.* Will Taft, Cincinnati, O. *Cor. Sec.* N. W. Williams, Xenia, O.

MISSOURI DENTAL ASSOCIATION—Meets on the first Tuesday of June, in St. Louis *Pres.* H. JUDD, St. Louis, *Sec.* W. H. EAMES, St. Louis.

MISSOURI VALLEY DENTAL SOCIETY.* Meets on the second Tuesday in July and January *Pres.* E. J. WOODBURY of Council Bluffs, Iowa *Sec. and Treas.* J. [illegible], Sanborn, Tabor, Iowa.

MERRIMAC VALLEY DENTAL SOCIETY—Meets semi annually first Tuesday of Oc and May.* *Pres.* A. LAWRENCE, Lowell. Mass. *Rec. Sec.* G. A. GERRY, of Lowell *Cor. Sec.* D. T. PORTER, Lawrence, Mass.

MEMPHIS DENTAL SOCIETY.—Meets on the first Tuesday of each month. *Pres* W. T. Arrington, *Sec.* V. F. Elliott. *Treas.* C. L. Harris.

NASHVILLE DENTAL ASSOCIATION—Meets on the second Tuesday of each month *Pres.* W. H. MORGAN, *Sec* J C. Ross, *Cor. Sec.* R RUSSELL

NEW YORK STATE DENTAL SOCIETY—Meets at Albany on the last Tuesday of July next *Pres.* A. WESTCOTT, of Syracuse. *Sec.* L. W. ROGERS, of Utica

NEW HAVEN DENTAL SOCIETY—Meets on the first Wednesday of each month at New Haven. *Pres.* J. T. METCALF. *Rec and Cor. Sec.* C. L. SMITH, of New Haven.

NEW LONDON COUNTY DENTAL SOCIETY—Meets monthly.* *Pres.* R W BROWNE. *Rec. Sec.* WM. ALLENDER

NORTHERN OHIO DENTAL ASSOCIATION—Meets Semi-annually, (first Tuesday of May and October ) in Cleveland. *Pres.* W. P. HORTON, *Rec. Sec.* H L. AMBLER, *Cor. Sec.* CHAS BUFFETT, Cleveland.

NORTHERN IOWA DENTAL ASSOCIATION—Meets annually. *Pres.* C. R. STERNEMAN Cedar Rapids. *Rec. Sec.* P. C. BRANCH, Dubuque.

OHIO DENTAL COLLEGE ASSOCIATION — Meets annually in March, at Cincinnati *Pres.* Geo H. Cushing, Chicago, Ill. *Rec Sec.* J TAFT, of Cincinnati.

OHIO STATE DENTAL ASSOCIATION.—Meets semi-annually, at Columbus. [Nex meeting—first Tuesday of December.] *Pres.* W P. HORTON, Cleveland, *Rec. Sec.* G. W. KEELY, Oxford, O. *Cor. Sec.* C. R. BUTLER, Cleveland, O

ODONTOGRAPHIC SOCIETY OF PENNSYLVANIA—Meets monthly in Philadelphia Next meeting, Wednesday, Sept 1st *Pres.* J. H. McQUILLEN. *Rec. Sec.* WM. H. HOWARD. *Cor. Sec.* T. C. STELLWAGEN.

ODONTOLOGICAL SOCIETY OF NEW YORK CITY—Meets the second Tuesday of each month * *Pres.* C. E. FRANCIS *Rec Sec.* C. F. IVES.

OLD COLONY DENTAL ASSOCIATION — Meets quarterly.* *Pres.* C. G. Davis. *Rec. Sec.* L. W. PUFFER, North Bridgewater, Mass. *Cor. Sec.* N C. FOWLER, Yarmouth, Mass.

PENNSYLVANIA ASSOCIATION OF DENTAL SURGEONS—Meets monthly in Phila. *Pres.* E. WILLIAMS. *Rec. Sec* JAMES TRUMAN *Cor. Sec.* E. T. DARBY.

PITTSBURG DENTAL ASSOCIATION—Meets first Tuesday of each month in Pittsburg. *Pres.* C. L. WESTENBURG *Rec. Sec.* J. D. WHITE

POUGHKEEPSIE DENTAL SOCIETY.—Meets monthly, *Pres.* J. H. Mann, *Sec.* H. F Clark.

ST. LOUIS DENTAL SOCIETY—Meets monthly. *Pres.* ISAAC COMSTOCK. *Sec.* R. J. POORE.

SUSQUEHANNA DENTAL ASSOCIATION—Meets semi-annually*. *Pres.* C. S. BECK, M. D. *Rec. Sec.* R. C. BURLEM.

SOCIETY OF DENTAL SURGEONS OF THE CITY OF NEW YORK—Meets semi-monthly in New York. *Pres.* B. W FRANKLIN. *Rec. Sec.* J. S. LATIMER. *Cor. Sec.* T. H. BURRAS.

SOUTHERN STATES DENTAL ASSOCIATION—Meets annually. Next meeting the 2d Wednesday in April, 1870, at New Orleans. *Pres.* W. T. ARRINGTON. *Rec. Sec* Prof. ANGELL, New Orleans

STATE DENTAL SOCIETY OF PENNSYLVANIA—Meets annually Third Tuesday in June.* Next meeting in Pittsburg, 1870. *Pres.* T. L Buckingham of Philadelphia. *Rec. Sec.* Geo. W. Neidich *Cor. Sec.* Samuel Welchens.

TENNESSEE DENTAL ASSOCIATION.—Meets semi-annually. *Pres.* W. T. Arrington, of Memphis, *Sec.* Wm M. R. Johns, of Somerville.

TUSCARAWAS VALLEY DENTAL SOCIETY.—The annual meeting in Nov. 1870. *Pres.* JOHN McKINLY, Uhricsville. *Sec.* L. E. DISNEY, Coshocton

WASHINGTON CITY DENTAL ASSOCIATION—Meets on the first Monday of each month. *Pres.* R. F. HUNT. *Sec.* J. C SMITH. *Librarian,* H. N. WADSWORTH.

WESTERN NEW YORK DENTAL ASSOCIATION—Meets semi-annually.* *Pres.* FRANK FRENCH, Rochester. *Sec* W. C. BARRETT, Warsaw.

**Place of meeting fixed by appointment*

### DISTRICT DENTAL SOCIETIES OF THE STATE OF NEW YORK.

*1ST DISTRICT DENTAL SOCIETY.—*Pres.* A. L. Northrop, New York City. *Sec.* W. C Horne, New York City.

2D DISTRICT DENTAL SOCIETY.—*Pres.* W. B. Hurd, Brooklyn. *Rec. Sec.* Wm. Jarvie Jr., Brooklyn. *Cor. Sec.* L S. Straw, Newburg

3D DISTRICT DENTAL SOCIETY.—Meets at Albany semi-annually and annually. *Pres* H. H Young, Troy. *Sec.* W. F. Winne, of Albany.

*4TH DISTRICT DENTAL SOCIETY.—*Pres.* F. C. Rich, Saratogo Springs. *Sec.* C. A Elmore, Fort Edwards.

*5TH DISTRICT DENTAL SOCIETY.—*Pres.* G. A. Foster, Utica *Sec.* Charles Barnes, Syracuse.

*6TH DISTRICT DENTAL SOCIETY.—*Pres.* S. H. McCall, Binghampton. *Sec.* F. B. DARBY, Elmira.

*7TH DISTRICT DENTAL SOCIETY.—*Pres.* A. G. COLEMAN, Canandagua. *Sec.* H. S. MILLER, Rochester.

TH DISTRICT DENTAL SOCIETY.—*Pres.* B. T. Whitney, Buffalo. *Rec. Sec.* W. C. Barrett, Warsaw. *Cor. Sec.* T. G. Lewis, Buffalo.

* Place and time of meeting fixed by appointment.

### EXCHANGES,

ST. LOUIS MEDICAL AND SURGICAL JOURNAL,
BOSTON MEDICAL AND SURGICAL JOURNAL,
THE PACIFIC MEDICAL AND SURGICAL JOURNAL SAN FRANCISCO.
BUFFALO MEDICAL AND SURGICAL JOURNAL.
PHILADELPHIA MEDICAL AND SURGICAL REPORTER.
CINCINNATI LANCET AND OBSERVER,
DENTAL COSMOS, PHILADELPHIA.
L'ART DENTAIRE, PARIS, FRANCE.
BRAITHWAITE'S RETROSPECT, N. Y.
THE DENTAL TIMES, PHILADELPHIA,
LADIES' REPOSITORY, CINCINNATI.
HALL'S JOURNAL OF HEALTH, N. Y,
CHICAGO MEDICAL EXAMINER.
XENIA TORCHLIGHT.
THE DETROIT REVIEW OF MEDICINE AND PHARMACY.
MEDICAL RECORD, N. Y.
NASHVILLE JOURNAL OF MEDICINE AND SURGERY,
AMERICAN ECLECTIC MEDICAL REVIEW, N. Y.
AMERICAN JOURNAL OF DENTAL SCIENCE, BALTIMORE.
THE UNITED STATES MEDICAL AND SURGICAL JOURNAL, CHICAGO,
NEW ORLEANS JOURNAL OF MEDICINE.
WESTERN JOURNAL OF MEDICINE. INDIANAPOLIS, IND.
AMERICAN AGRICULTURIST, N. Y.
DENTAL OFFICE AND LABORATORY, PHILA.
BOSTON JOURNAL OF CHEMISTRY.
CINCINNATI MEDICAL REPERTORY.
CHICAGO MEDICAL INVESTIGATOR.
JOURNAL OF APPLIED CHEMISTRY, N. Y.
DRUGGISTS' CIRCULAR AND CHEMICAL GAZETTE, NEW YORK.
DER ZAHNARZT, LEIPZIG.
DEUTSCHE VIERTELJAHRSSCHRIFT, NUREMBURG.
CANADA JOURNAL OF DENTAL SCIENCE, MONTREAL.
GUARDIAN OF HEALTH AND EDUCATION, BOSTON.
HARDWICKE'S SCIENCE GOSSIP, LONDON, ENGLAND.
PACKARD'S MONTHLY, NEW YORK
POPULAR SCIENCE REVIEW, LONDON, ENGLAND.
MISSOURI DENTAL MONTHLY ST. LOUIS.
MEDICAL BULLETIN, BALTIMORE, MD.
ECLECTIC MEDICAL JOURNAL, CINCINNATI.
TRANSACTIONS, COLLEGE OF PHYSICIANS, PHILADELPHIA.

### MEETINGS.

The twenty-sixth annual meeting of the Mississippi Valley Dental Society will be held in the lecture room of the Ohio Dental College, on March 2d, prox. at 10 o'clock, A. M., when we hope to meet all the members, and all others who can possibly make it convenient to be present.

The meetings of this society are always of very great interest. We would call attention to the subjects presented for consideration by the Executive committee, on page 40. Let every one who contemplates being present make some preparation upon each of the subjects, so that every one may feel that he has done good to others, as well as received good himself. It is full time that those who are always receiving, and never giving should begin to balance accounts. T.

### BILLS.

We send out with this number, bills for the present volume, also bills for all arrearages ; to all of which we trust our subscribers will give prompt attention.

A strong inducement to do this will be found on our special notice page.

Any who have fulfilled the requirements of that proposition, as well as those who may by the first of March next, will, by notifying us, receive a copy of the Engagement Book. T.

The annual meeting of the Ohio Dental College Association will be held in the College, Tuesday, March 1st, at 10 o'clock, A. M. As matters of importance will come before the meeting, it is desirable that every member be present. We hope all feel interested enough in the institution to take part in its management. T.

### ACKNOWLEDGMENT.

We received, a short time ago, $100 worth of very fine teeth from S. S. White, as a contribution to the Ohio College of Dental Surgery. Mr. W. will please accept the thanks of every one connected with the College. We regard this, with other things of the same kind, as substantial appreciation of the Institution.

### CORRECTIONS.

There were a few misprints in the article "Miscellanies," Dec. No.—page 417, 14th line from top, "with the understanding," should read without the understanding. Page 419, first line, new paragraph, for "But these are pictures," it should be, there are pictures.

In the December No. of the Register, page 516, the article credited to Dr. Parker, should have been credited to Dr. Wm. P. Barker, Grand Rapids, Mich.

### SPECIAL NOTICES.

ANOTHER PROPOSITION.—We will send to every one of our subscribers, who will send us his subscription to the DENTAL REGISTER for the present year, 1870, together with any back subscription he may owe, one copy of the Dentist's Engagement Book, for 1870. This proposition is to every one who pays up to Jan., 1871. J. TAFT.

FOR SALE.—A Dental office, in Boston, to be disposed of immediately, with all the instruments and fixtures of the office, and the tools and appliances of the laboratory. The office is splendidly located. The practice is large, about $4,000 per year. The rent of the office is merely nominal.

Address, DENTIST, Station A, Boston, Mass.

FOR SALE.—Dental Practice, Office, Lease of House, etc. Located in the city of Louisville, Ky. The office is well furnished. The Laboratory has steam engine complete, lathes, etc., and every thing necessary for a large business. Established in 1865, and has a business of eight thousand a year. Rent low. Cause of selling, retiring from business.

Address, DR. YATES, 103 Second St.

### CONES.

We have for some time been using Felt Cones for polishing purposes, manufactured by JOHNSTON & LUND, of Philadelphia.

They serve a most valuable purpose, and no one who becomes accustomed to their use, would be satisfied without them, unless there is something better that has not been made public.

Our advice is, give them a trial.

AMERICAN DENTAL ASSOCIATION—Meets on the first Tuesday in August next year at Nashville, Tenn. *Pres.* H. JUDD, St. Louis. *Rec. Sec.* M. S. DEAN, Chicago.

AMERICAN DENTAL CONVENTION.—Meets in New York, Sept. 1870. *Pres.* Dr. J. G. AMBLER. *Sec.* and *Treas* Dr. J. H. SMITH, New Haven Conn.

*List of Societies represented in the American Dental Association.*

AMERICAN DENTAL CONVENTION—Meets annually. *Pres.* J. M. CROWELL of New York, *Rec.* and *Cor Sec* J. S. LATIMER, of New York.

AMERICAN ACADEMY OF DENTAL SCIENCE—Meets at Boston, Mass. *Pres.* DANIEL HARWOOD, M. D., of Boston. *Sec.* E. N HARRIS, D. D. S., of Boston.

BROOKLYN DENTAL SOCIETY—Meets semi-monthly. *Pres.* C. D. Cook. *Rec. Sec.* E L. CHILDS. *Cor. Sec.* WM. JARVIE. Jr.

BUCKS COUNTY DENTAL ASSOCIATION, (PA.) Meets semi-annually on the first Monday in May and November. Next meeting at Newton, in November. *Pres.* H. P. Yerkes, *Sec.* G. W. Adams.

BUFFALO DENTAL SOCIETY—Meets monthly at Buffalo. *Pres.* B. F. Whitney, *Rec. Sec.* S. A. FREEMAN.

BOSTON DENTAL INSTITUTE. Meets first Tuesday of each month. *Pres.* D. G. Williams. *Cor. Sec.* D. S. Dickerman.

CUMBERLAND VALLEY DENTAL SOCIETY—Meets semi-annually.* *Pres.* J. L. SUESSEROTT, of Chambersburg. *Sec.* GEO. W. NEIDICH, of Carlisle, Pa.

CINCINNATI DENTAL SOCIETY—Meets semi-monthly at Cincinnati. *Pres.* Geo. WATT, *Rec. Sec.* Wm. TAFT.

CENTRAL OHIO DENTAL ASSOCIATION—Meets Annually on the second Tuesday of July. *Pres.* S. P. HARRINGTON, Newark. *Sec.* G. W. DECAMP Mansfield, O.

CHICAGO DENTAL SOCIETY—Meets monthly at Chicago. *Pres.* J. H. YOUNG. *Rec. and Cor. Sec.* C. R E. KOCH.

CONNECTICUT VALLEY DENTAL ASSOCIATION—Meets semi-annually, second Tuesday of May and October, *Pres.* A. A. HOWLAND, Barre, Vt. *Rec. Sec.* W. H. JONES, of Boston, Mass.

CONNECTICUT STATE DENTAL ASSOCIATION—*Pres.* SAML. MALLETT, New Haven. *Cor. Sec.* JOHN CODY, Hartford. *Rec. Sec.* Dr. POWERS, Hartford.

CHARLESTON DENTAL ASSOCIATION.*—*Prest.* I. B. PATRICK. *Sec. and Treas.* T. F. CHUPEIN.

DELAWARE DENTAL ASSOCIATION—Meets semi-annually. *Pres.* E, W. HAINES, Newark. *Sec.* C. R. JEFFRIES, Wilmington.

EAST TENNESSEE DENTAL ASSOCIATION—Meets annually at Knoxville. Tenn. *Pres.* J. A. CRAZIER of Knoxville *Rec. Sec.* W. F. FOWLER, Tenn.

FOREST CITY SOCIETY OF DENTAL SURGEONS. *Pres.* B. Strickland, Cleveland. *Rec. Sec.*, H. L. Ambler, Cleveland. *Cor. Sec.*, Chas. Buffett, Cleveland.

HARRIS DENTAL ASSOCIATION OF LANCASTER, Pa—Next meeting at Columbia, on Thursday the [illegible]th of Nov. next. *President,* SAM'L. WELCHENS. *Sec.* WM. NICHOLS AMER.

HUDSON RIVER ASSOCIATION OF DENTAL SURGEONS—Meets on the second. Wednesdays of May, September and January. *Pres.* L. S. STRAW, Newburg N.Y. *Rec. Sec.* T. W. DUBOIS, Pougkeepsie, N. Y.

HUDSON VALLEY DENTAL ASSOCIATION—meets monthly at Troy, N. Y. *Pres.* C. H. JENKINS, *Rec. Sec.* E. J. YOUNG, *Cor. Sec.* S. D. FRENCH.

ILLINOIS STATE DENTAL SOCIETY—Meets annually. *Pres.* M S. Dean, of Chicago. *Sec* C. S. SMITH, of Springfield.

INDIANA STATE DENTAL SOCIETY—Meets annually at Indianapolis on the last Tuesday of June. *Pres.* J. F. Johnston, of Indianapolis. *Rec. and Cor. Sec* S. B. Brown.

IOWA STATE DENTAL SOCIETY—Meets annually. *Next meeting at Iowa City, on the second Tuesday of July. *Pres.* J. HARDMAN, of Muscatine. *Rec. Sec.* A. B MASON, of Waterloo. *Cor Sec.* J. P. WILSON, of Burlington.

LAKE ERIE DENTAL ASSOCIATION.—Meets Annually.* *Pres.* B.T. WHITNEY, Buffalo N. Y., *Sec.* W. E. MAGILL, Erie, Pa.

LEBANON VALLEY DENTAL ASSOCIATION—*Prest.* W. K. BRENIZER, *Sec.* S. H. GUILFORD.

MAD RIVER VALLEY DENTAL SOCIETY—Meets Semi annually on the first Tuesday of April and Oct. Next meeting at Springfield, O. *Pres.* J. H. PAINE, Middletown, O. *Sec.*, S. B. TIZZARD, Troy, O.

MARYLAND ASSOCIATION OF DENTISTS—Meets the last Thursday of every month. *Pres.*, J. B. BEAN, *Sec.*, S M. FIELD.

MASSACHUSETTS DENTAL SOCIETY—Meets monthly. *Pres.*, T. H. Chandler, *Rec. Sec.* A. Brown, *Cor. Sec.* E. Blake.

MICHIGAN DENTAL SOCIETY—Meets annually second Tuesday of Oct. at Detroit. *Pres.* George Leary, of Adrian, *Rec. and Cor. Sec.* W. H. Jackson, of Ann Arbor.

MUSKINGUM VALLEY DENTAL ASSOCIATION—Meets on first Monday of each month at Zanesville, O. *Pres.* W. M. Herriot, of Zanesville, O., *Sec.* W. W. Chapelear, Zanesville, O.

MISSISSIPPI VALLEY DENTAL ASSOCIATION—Meets annually at Cincinnati, on the first Wednesday in March. *Pres.* A. M. Moore, Lafayette, Ind. *Rec. Sec.* Will Taft, Cincinnati, O. *Cor. Sec.* N. W. Williams, Xenia, O.

MISSOURI DENTAL ASSOCIATION—Meets on the first Tuesday of June, in St. Louis *Pres.* H. JUDD. St. Louis, *Sec* W. H. EAMES, St. Louis.

MISSOURI VALLEY DENTAL SOCIETY.* Meets on the second Tuesday in July and January. *Pres.* E. J. WOODBURY of Council Bluffs, Iowa *Sec. and Treas.* J. [illegible], Sanborn, Tabor, Iowa.

MERRIMAC VALLEY DENTAL SOCIETY—Meets semi annually first Tuesday of Oc and May.* *Pres.* A. LAWRENCE, Lowell. Mass. *Rec. Sec.* G. A. GERRY, of Lowell *Cor. Sec.* D. T. PORTER, Lawrence, Mass.

MEMPHIS DENTAL SOCIETY.—Meets on the first Tuesday of each month. *Pres* W. T. Arrington, *Sec.* V. F. Elliott. *Treas.* C. L. Harris.

NASHVILLE DENTAL ASSOCIATION—Meets on the second Tuesday of each month *Pres.* W. H. MORGAN, *Sec* J C. ROSS, *Cor. Sec.* R RUSSELL.

NEW YORK STATE DENTAL SOCIETY—Meets at Albany on the last Tuesday of July next. *Pres.* A. WESTCOTT, of Syracuse. *Sec.* L. W. ROGERS, of Utica

NEW HAVEN DENTAL SOCIETY—Meets on the first Wednesday of each month at New Haven. *Pres.* J. T. METCALF, *Rec and Cor. Sec.* C. L. SMITH, of New Haven.

NEW LONDON COUNTY DENTAL SOCIETY—Meets monthly.* *Pres.* R. W. BROWNE. *Rec. Sec.* WM. ALLENDER

NORTHERN OHIO DENTAL ASSOCIATION—Meets Semi-annually, (first Tuesday of May and October ) in Cleveland. *Pres.* W. P. HORTON, *Rec. Sec.* H L. AMBLER, *Cor. Sec.* CHAS. BUFFETT, Cleveland.

NORTHERN IOWA DENTAL ASSOCIATION—Meets annually, on the 2d Tuesday in June, 1870. Next meeting at Waterloo. *Pres.* E. S. CLARK, Dubuque. *Rec. Sec.* J. Z. ABBOTT, Manchester. *Cor. Sec.* A. B. MASON, Waterloo.

OHIO DENTAL COLLEGE ASSOCIATION—Meets annually in March, at Cincinnati *Pres.* Geo H. Cushing, Chicago, Ill. *Rec Sec.* J. TAFT, of Cincinnati.

OHIO STATE DENTAL ASSOCIATION.—Meets semi-annually, at Columbus. [Nex meeting—first Tuesday of December.] *Pres.* W. P. HORTON, Cleveland, *Rec. Sec.* G. W. KEELY, Oxford, O. *Cor. Sec.* C. R. BUTLER, Cleveland, O

ODONTOGRAPHIC SOCIETY OF PENNSYLVANIA—Meets monthly in Philadelphia Next meeting, Wednesday, Sept. 1st. *Pres.* J. H. McQUILLEN. *Rec. Sec.* WM. H. HOWARD. *Cor. Sec.* T. C. STELLWAGEN.

ODONTOLOGICAL SOCIETY OF NEW YORK CITY—Meets the second Tuesday of each month.* *Pres.* C E. FRANCIS. *Rec Sec.* C. F. IVES.

OLD COLONY DENTAL ASSOCIATION —Meets quarterly.* *Pres.* C. G. Davis. *Rec. Sec.* L. W. PUFFER, North Bridgewater, Mass. *Cor. Sec.* N. C. FOWLER, Yarmouth, Mass.

PENNSYLVANIA ASSOCIATION OF DENTAL SURGEONS—Meets monthly in Phila. *Pres.* E. WILLIAMS. *Rec. Sec* JAMES TRUMAN *Cor. Sec.* E. T. DARBY.

PITTSBURG DENTAL ASSOCIATION—Meets first Tuesday of each month in Pittsburg. *Pres.* C. L. WESTENBURG *Rec. Sec.* J. D. WHITE.

POUGHKEEPSIE DENTAL SOCIETY.—Meets monthly, *Pres.* J. H. Mann, *Sec.* H. F Clark.

ST. LOUIS DENTAL SOCIETY—Meets monthly. *Pres.* ISAAC COMSTOCK. *Sec.* R. J. POORE.

SUSQUEHANNA DENTAL ASSOCIATION—Meets semi-annually*. *Pres.* C. S. BECK M. D. *Rec. Sec.* R. C. BURLEM.

SOCIETY OF DENTAL SURGEONS OF THE CITY OF NEW YORK—Meets semi-monthly in New York. *Pres.* B. W FRANKLIN. *Rec. Sec.* J. S. LATIMER. *Cor. Sec.* T. H. BURRAS.

SOUTHERN STATES DENTAL ASSOCIATION—Meets annually. Next meeting the 2d Wednesday in April, 1870, at New Orleans. *Pres.* W. T. ARRINGTON. *Rec. Sec.* Prof. ANGELL, New Orleans

STATE DENTAL SOCIETY OF PENNSYLVANIA.—Meets annually Third Tuesday in June.* Next meeting in Pittsburg, 1870. *Pres.* T. L. Buckingham of Philadelphia. *Rec. Sec.* Geo. W. Neidich. *Cor. Sec.* Samuel Welchens.

TENNESSEE DENTAL ASSOCIATION.—Meets semi-annually. *Pres.* W. T. Arrington of Memphis *Sec.* Wm M. R. Johns, of Somerville.

TUSCARAWAS VALLEY DENTAL SOCIETY.—The annual meeting in Nov. 1870. *Pres.* JOHN MCKINLY, Uhricsville. *Sec.* L. E. DISNEY, Coshocton.

WASHINGTON CITY DENTAL ASSOCIATION—Meets on the first Monday of each month. *Pres.* R. F. HUNT. *Sec.* J. C SMITH. *Librarian*, H. N. WADSWORTH.

WESTERN NEW YORK DENTAL ASSOCIATION—Meets semi-annually.* *Pres.* FRANK FRENCH, Rochester. *Sec* W. C. BARRETT, Warsaw.

**Place of meeting fixed by appointment*

### DISTRICT DENTAL SOCIETIES OF THE STATE OF NEW YORK.

*1ST DISTRICT DENTAL SOCIETY.—*Pres.* A. L. Northrop, New York City. *Sec.* W. C Horne, New York City.

2D DISTRICT DENTAL SOCIETY.—*Pres.* W. B. Hurd, Brooklyn. *Rec. Sec.* Wm. Jarvie Jr., Brooklyn. *Cor. Sec.* L S. Straw, Newburg

3D DISTRICT DENTAL SOCIETY.—Meets at Albany semi-annually and annually. *Pres* H. H. Young, Troy. *Sec.* W. F. Winne, of Albany.

*4TH DISTRICT DENTAL SOCIETY.—*Pres.* F. C. Rich, Saratogo Springs. *Sec.* C. A Elmore, Fort Edwards.

*5TH DISTRICT DENTAL SOCIETY.—*Pres.* G. A. Foster, Utica *Sec.* Charles Barnes, Syracuse.

*6TH DISTRICT DENTAL SOCIETY.—*Pres.* S. H. McCall, Binghampton. *Sec.* F. B. Darby, Elmira.

*7TH DISTRICT DENTAL SOCIETY.—*Pres.* A. G. Coleman, Canandagua. *Sec.* H. S. Miller, Rochester.

TH DISTRICT DENTAL SOCIETY.—*Pres.* B. T. Whitney, Buffalo. *Rec. Sec.* W. C. Barrett, Warsaw. *Cor. Sec.* T. G. Lewis, Buffalo.

* Place and time of meeting fixed by appointment.

### EXCHANGES,

ST. LOUIS MEDICAL AND SURGICAL JOURNAL
BOSTON MEDICAL AND SURGICAL JOURNAL,
THE PACIFIC MEDICAL AND SURGICAL JOURNAL SAN FRANCISCO.
BUFFALO MEDICAL AND SURGICAL JOURNAL.
PHILADELPHIA MEDICAL AND SURGICAL REPORTER.
CINCINNATI LANCET AND OBSERVER,
DENTAL COSMOS, PHILADELPHIA.
L'ART DENTAIRE, PARIS, FRANCE.
BRAITHWAITE'S RETROSPECT, N. Y.
THE DENTAL TIMES, PHILADELPHIA,
LADIES' REPOSITORY, CINCINNATI.
HALL'S JOURNAL OF HEALTH, N. Y,
CHICAGO MEDICAL EXAMINER.
XENIA TORCHLIGHT.
THE DETROIT REVIEW OF MEDICINE AND PHARMACY.
MEDICAL RECORD, N. Y.
NASHVILLE JOURNAL OF MEDICINE AND SURGERY,
AMERICAN ECLECTIC MEDICAL REVIEW, N. Y.
AMERICAN JOURNAL OF DENTAL SCIENCE, BALTIMORE.
THE UNITED STATES MEDICAL AND SURGICAL JOURNAL. CHICAGO,
NEW ORLEANS JOURNAL OF MEDICINE.
WESTERN JOURNAL OF MEDICINE. INDIANAPOLIS, IND.
AMERICAN AGRICULTURIST, N. Y.
DENTAL OFFICE AND LABORATORY, PHILA.
BOSTON JOURNAL OF CHEMISTRY.
CINCINNATI MEDICAL REPERTORY.
CHICAGO MEDICAL INVESTIGATOR.
JOURNAL OF APPLIED CHEMISTRY, N. Y.
DRUGGISTS' CIRCULAR AND CHEMICAL GAZETTE, NEW YORK.
DER ZAHNARZT, LEIPZIG.
DEUTSCHE VIERTELJAHRSSCHRIFT, NUREMBURG.
CANADA JOURNAL OF DENTAL SCIENCE, MONTREAL.
GUARDIAN OF HEALTH AND EDUCATION, BOSTON.
HARDWICKE'S SCIENCE GOSSIP, LONDON. ENGLAND.
PACKARD'S MONTHLY, NEW YORK.
POPULAR SCIENCE REVIEW, LONDON, ENGLAND.
MISSOURI DENTAL MONTHLY ST. LOUIS.
MEDICAL BULLETIN, BALTIMORE, MD.
ECLECTIC MEDICAL JOURNAL, CINCINNATI.
TRANSACTIONS, COLLEGE OF PHYSICIANS, PHILADELPHIA.

